# The RNA m^6^A writer WTAP in diseases: structure, roles, and mechanisms

**DOI:** 10.1038/s41419-022-05268-9

**Published:** 2022-10-07

**Authors:** Qibo Huang, Jie Mo, Zhibin Liao, Xiaoping Chen, Bixiang Zhang

**Affiliations:** Hubei Key Laboratory of Hepato-Pancreato-Biliary Diseases; Hepatic Surgery Center, Tongji Hospital, Tongji Medical College, Huazhong University of Science and Technology; Clinical Medicine Research Center for Hepatic Surgery of Hubei Province; Key Laboratory of Organ Transplantation, Ministry of Education and Ministry of Public Health, Wuhan, Hubei 430030 P.R. China

**Keywords:** Oncogenes, Methylation

## Abstract

N6-methyladenosine (m^6^A) is a widely investigated RNA modification in studies on the “epigenetic regulation” of mRNAs that is ubiquitously present in eukaryotes. Abnormal changes in m^6^A levels are closely related to the regulation of RNA metabolism, heat shock stress, tumor occurrence, and development. m^6^A modifications are catalyzed by the m^6^A writer complex, which contains RNA methyltransferase-like 3 (METTL3), methyltransferase-like 14 (METTL14), Wilms tumor 1-associated protein (WTAP), and other proteins with methyltransferase (MTase) capability, such as RNA-binding motif protein 15 (RBM15), KIAA1429 and zinc finger CCCH-type containing 13 (ZC3H13). Although METTL3 is the main catalytic subunit, WTAP is a regulatory subunit whose function is to recruit the m^6^A methyltransferase complex to the target mRNA. Specifically, WTAP is required for the accumulation of METTL3 and METTL14 in nuclear speckles. In this paper, we briefly introduce the molecular mechanism of m^6^A modification. Then, we focus on WTAP, a component of the m^6^A methyltransferase complex, and introduce its structure, localization, and physiological functions. Finally, we describe its roles and mechanisms in cancer.

## Facts

- N6-methyladenosine RNA modification (m^6^A) is one of the most abundant modifications in eukaryotic mRNA, which plays an important role in cancer initiation and progression.
- m^6^A methylation is catalyzed by a multicomponent methyltransferase complex including: METTL3, METTL14, WTAP, METTL16, KIAA1429, RBM15, RBM15B, ZC3H13. WTAP serves as an essential regulatory subunit in methyltransferase which recruits m6A methyltransferase complex to the target mRNA.
- WTAP plays dual roles in cancer either as an oncogene or as a tumor suppressor. It might regulate cancer though m6A methylation or other signaling pathways.

## Open questions

- How does WTAP recruit methyltransferase complex to the target mRNA?
- What determines WTAP localization and in what condition WTAP forms up complexes as WTAP-BCLAF1-THRAP3, WT1-WTAP, or METTL3-METTL14-WTAP?

## Background

Epigenetics is a branch of genetics that investigates heritable changes in gene expression without changes in the nucleotide sequence [1, 2]. Epigenetic regulation has been observed in the context of DNA methylation [3], histone modifications [4], chromatin remodeling [5], transcriptional control [6], noncoding RNAs [7], and cancer immunotherapy [8]. Posttranscriptional modifications, including m1A [9], m5C [10], and m^6^A [11], are abundant and significant, especially m^6^A modifications, because they are considered the most abundant internal modification in eukaryotes [12], with approximately 25% of mRNAs carrying at least one m^6^A site [13, 14]. m^6^A modifications can be added not only to mRNAs but also to rRNAs, small nucleolar RNAs (snRNAs), and microRNAs [7, 15]. m^6^A modification affects RNA export, leads to spliced pre-mRNAs, and impacts RNA translation and stability [16]. Abnormal regulation of m^6^A has been observed in cancers, and its role as an oncogene or tumor suppressor depends on the cellular environment [17, 18].

The main methyltransferases are METTL3, METTL14, and WTAP, which form the m^6^A methyltransferase complex (MTC). The m^6^A level is largely dependent on the MTC. Numerous studies have revealed that the m^6^A level is of great concern in heart failure [19], testosterone synthesis [20], liver steatosis [21], and different cancers [22, 23]. The m^6^A modification plays a dual role in cancer biology and is important for the recognition of cancer progression and cancer therapy [24]. To provide a more comprehensive understanding of m^6^A methyltransferase, we focused on WTAP, a constituent of the m^6^A methyltransferase complex.

WTAP was first identified as a splicing factor and then confirmed to be the third component of methyltransferase [14, 25, 26]. In addition, WTAP fulfils several biological functions, including embryo development, cell cycle progression, cell differentiation, pre-mRNA splicing, and antiviral responses. In this review, we first describe the biological functions of WTAP in detail. Then, we focus on the role of WTAP in cancers either dependent or independent of METTL3-METTL14 methyltransferase and summarize the specific mechanisms of WTAP in tumorigenesis and development.

## Molecular mechanism of m^6^A modification

m^6^A is a widely investigated RNA modification in studies on “epigenetic regulation” [27, 28]. The m^6^A RNA modification accounts for 80% of all RNA modifications related to pre-mRNA splicing, miRNAs, lncRNAs, circRNA processing, translation efficiency, and mRNA stability [29]. m^6^A is a dynamic, reversible posttranscriptional modification. The residues of adenosine at the N6 position are localized in the 3ʹ untranslated region (UTR) of the mRNA or close to the termination codon [30, 31]. This modification can occur in different biological processes and is mediated by corresponding enzymes termed “writers,” “erasers,” and “readers” [32].

Methyltransferase-like protein 3 (METTL3) and S-adenosylmethionine (SAM)-binding protein [33] are the most significant components of the methyltransferase complex [34–38]. Methyltransferase-like protein 14 (METTL14) colocalizes with METTL3 in nuclear speckles at a 1:1 ratio [39–43], where it stabilizes the m^6^A methyltransferase complex (MTC) and recognizes specific RNA sequences (RRACH) [30, 44]. WTAP recruits METTL3 and METTL14 into nuclear speckles (associated with mRNA export) and is crucial for this unique localization [14, 25, 26]. Furthermore, RNA-binding motif protein 15 (RBM15) can bind to WTAP and recruit the MTC to specific RNA sites for m^6^A modification [45]. This process is important for the control of m^6^A-promoted X-chromosome inactivation in humans [46]. Zinc finger CCCH-type containing 13 (ZC3H13) interacts with WTAP to retain the MTC in nuclear speckles via its LC domain and thereby promotes its function [47, 48]. Other m^6^A writers have been revealed in recent years, including METTL16, METTL5, VIRMA, and ZCCHC4 [49–53].

After the “writers” mark the target mRNA, “reader” proteins, such as YT521-B homology (YTH) domain-containing protein [54–62], eukaryotic translation initiation factor 3 (eIF3) [63], the IGF2 mRNA binding protein (IGF2BP) family [64–67], and the heterogeneous nuclear ribonucleoprotein (HNRNP) protein family [68, 69], decode m^6^A methylation to generate signals for nuclear export, translation, RNA splicing, RNA stabilization, and decay [70].

Fat and obesity-related protein (FTO) [71–73] and alkB homolog 5 (ALKBH5) [74–76] are two essential enzymes for demethylation. “Erasers” are involved in building up the dynamic, reversible modification with “Writers” and “Readers” [77].

**Fig. 1:**
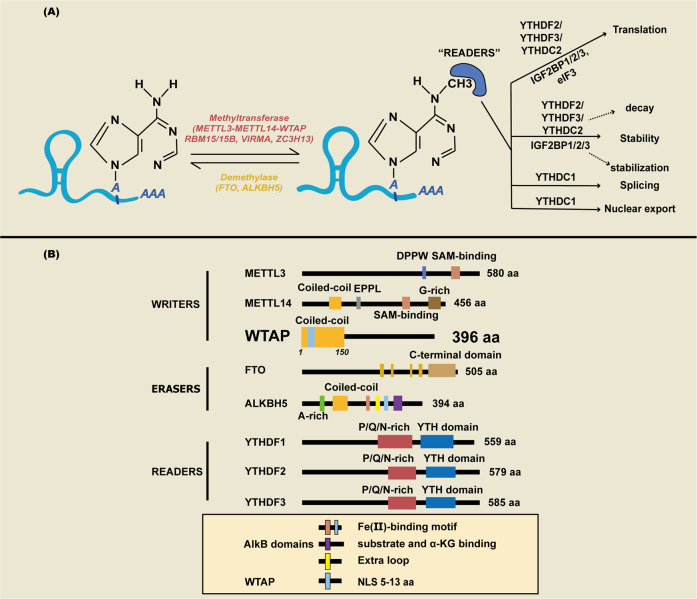
Mechanism of m6A and fuctional domais in m^6^A methyltransferase. **A** The dynamic molecular mechanism of m^6^A modification. m^6^A is installed by “writers” (METTL3/14, WTAP, RBM15/15B, VIRMA, and ZC3H13), removed by “erasers” (FTO, ALKBH5, and ALKBH3), and recognized by “readers” (YTHDC1/2, YTHDF1/2/3, IGF2BP1/2/3, HNRNP, and eIF3). **B** Functional domains in m^6^A writer, eraser, and reader proteins.

In general, m^6^A modification is an abundant and powerful epigenetic modification in eukaryotes. If one key enzyme is disordered, this dynamic modification is disrupted, which impacts human diseases (Table 1, Fig. 1).

**Table 1:** Summary of m^6^A modification enzymes.

Components	Enzymes	Intracellular localization	Biological functions	References
*WRITERS*	*METTL3*	Cytoplasm, Nucleus, Nuclear speckles	m^6^A methyltransferase, DNA damage responses, DNA‒RNA hybrid, Cancer cell proliferation, Cell cycle progression and survival, Cancer cell resistance to radiotherapy and cisplatin	[35–38]
	*METTL14*	Nucleus	m^6^A methyltransferase, mRNA degradation or stabilization, LncRNA stabilization, pre-mRNA splicing, mRNA exportation, mRNA turnover in tumor proliferation, Metastasis, Self-renewal and tumor-initiating capacity	[41–44]
	*WTAP*	Cytoplasm, Nucleus, Nuclear speckles	m^6^A methyltransferase, Embryo development, Cell cycle progression and differentiation, Pre-mRNA splicing, Antiviral responses, Alternative splicing	[78, 82–84, 86, 87, 98, 100, 101]
	*RBM15/ ZC3H13/ VIRMA*	Nuclear speckles, Nucleus, Nuclear envelope, Nuclear membrane	m^6^A methyltransferase, Proliferation, invasion, migration, and apoptosis, Anchoring the m^6^A regulatory complex in the nucleus, Controls mouse embryonic stem cell self-renewal	[45, 48, 51, 70]
	*ZCCHC4*	Nucleus, Cytoplasm	Methylates human 28 S rRNA, Interacts with a subset of mRNAs, Related to global translation, Cell proliferation	[52]
	*METTL5*	Nucleus, Cell junction	m^6^A modification of 18 S rRNA, Promotes translation initiation, S6K activation, and cancer cell growth	[50, 53]
	*METTL16*	Nucleus, Cytoplasm	m^6^A modification of U6 snRNA, lncRNAs, and introns of pre-mRNAs	[49]
*ERASERS*	*FTO*	Cytoplasm, Nucleus, Nuclear speckles	Demethylation of m^6^A and m1A, Regulation of mRNA splicing and cell differentiation	[71–73]
	*ALKBH5*	Nuclear speckles	m^6^A demethylation, Participates in the regulation of mRNA nuclear export and mouse sperm development, Reduces tumoral proliferative, migration, and invasion activities	[74–76]
*READERS*	*YTHDF2/3*	Nucleus, Cytoplasm	mRNA stabilization/degradation, Regulates mRNA clearance, Regulates cancer cell proliferation, invasion and migration	[54, 58, 59, 83]
	*YTHDC1*	Nucleus, Nuclear speckles	Binds m^6^A-modified pre-mRNAs and mRNAs, and facilitates exon inclusion, splicing, mRNA nuclear-cytoplasmic export	[55, 60, 61]
	*IGF2BP1–3*	Cytoplasm, Nucleus	Recognizes m^6^A through K homology domains and facilitates m6A-modified mRNA stabilization and protein translation	[64–67]
	*YTHDC2*	Cytoplasm	Regulates mRNA translation or decay and mouse spermatogenesis	[56]
	*YTHDF1*	Cytoplasm	Selectively recognizes m^6^A-modified mRNA, Promotes ribosome loading of m^6^A-modified mRNA, Interacts with initiation factors to facilitate translation initiation	[57, 62]
	*hnRNPC/hnRNPG*	Nucleus	Regulates mRNA structure and alternative splicing	[69]

## Overview of WTAP

### Structure and cellular localization of WTAP

Wilms’ tumor 1-associating protein (WTAP) is encoded on human chromosomal region 6q25.3 [78]. WTAP is a 44 kDa protein that contains 396 amino acids and is encoded by the human homolog of FL (2)d [79]. WTAP localizes to both the nucleus and cytoplasm [25, 80]. WTAP is a key component in m6A modification, forming a complex with VIRMA, CBLL1, ZC3H13 (KIAA0853), RBM15/15B, and METTL3/14 [80]. WTAP contains an extended N-terminal coiled-coil region followed by an unstructured C-terminal part [81] (Fig. 1B). WTAP regulates the localization of the stable heterodimer core complex of METTL3/14 into nuclear speckles through amino acids 5–13 of the nuclear localization signal (NLS) (-PLPKKVRL- to -PLPGGVGL-) at its N-terminus [81]. Notably, the N-terminal coiled-coil region (1–150 amino acids) that contains the NLS is the binding surface of METTL3, which links to the helical structure at the N-terminus of METTL3, called the leader helix (LH) [81]. Although WT1 was found to interact with WTAP, it was confirmed that WT1 was dispensable for the regulation of m^6^A modification by WTAP [25] (Fig. 1B).

### Biological functions of WTAP

#### Embryo development

In mice, WTAP plays an essential role in embryonic development. WTAP knockout embryos exhibit proliferative failure [82], and heterozygous mice die at embryonic day 10.5 [83]. In pigs, WTAP knockdown reduced the blastocyst rate and total m^6^A levels [84].

#### Cell cycle progression and differentiation

**Fig. 2:**
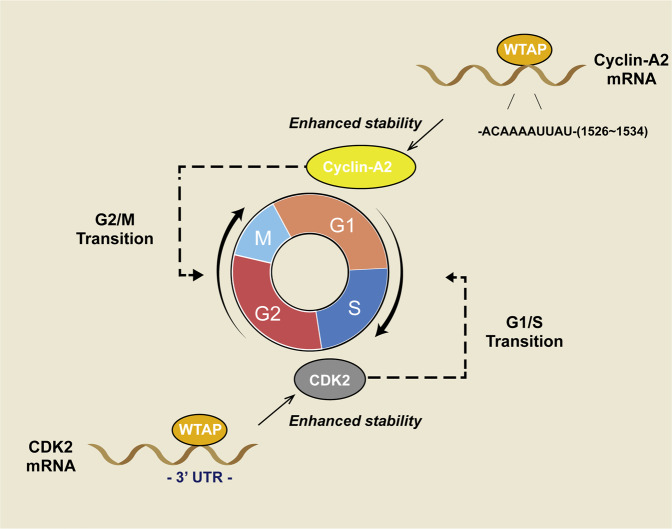
The function of WTAP in cell cycle transition. In keratinocytes and renal cell carcinoma cells, WTAP enhances the stability of the CDK2 mRNA by directly binding to its 3’-UTR. In human umbilical vein endothelial cells (HUVECs), WTAP stabilizes cyclin-A2 mRNA by binding to its AUUUA motif ACAAAUUAU, which corresponds to the 3ʹ UTR (1526–1534). These findings indicated that WTAP promotes the G1/S transition and the G2/M transition.

Cell proliferation and differentiation are the foundation of growth, development, reproduction, and heredity in organisms [85]. In human umbilical vein endothelial cells (HUVECs), decreased WTAP levels induced cell cycle arrest in the G2 phase. At the same time, the protein levels of cyclin-A2, B1, B2, and CDC20, which are related to the cell cycle [86, 87], were significantly decreased [82]. Mechanistically, WTAP stabilizes cyclin-A2 mRNA by binding to its AUUUA motif ACAAAUUAU, which corresponds to the 3ʹ UTR (1526–1534) [82]. These findings indicated that WTAP promotes the G2/M transition in HUVECs (Fig. 2) [82].

**Fig. 3:**
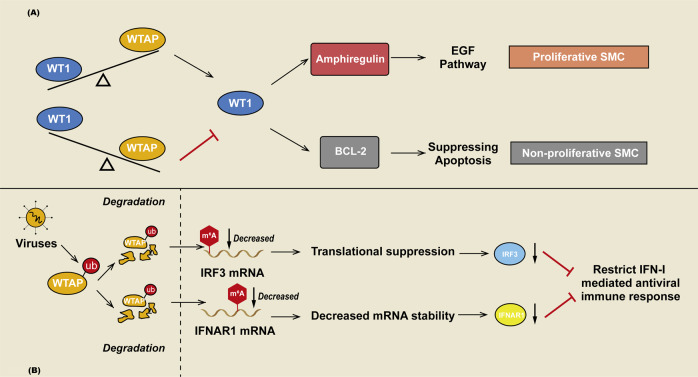
**A** Model of the mechanism through which WTAP regulates SMC proliferation. The balance between WTAP and WT1 influences the state of SMCs. When the expression of WTAP is reduced, WT1-mediated transcriptional events proceed. Amphiregulin is a direct transcriptional target of WT1 that drives SMC proliferation by upregulating the EGF pathway. Thus, SMCs switch to a proliferative state. When the balance of WTAP and WT1 is reversed, WT1-mediated transcription may be blocked, and the transcription of Bcl-2, which is suppressed by WT1, is activated. SMC apoptosis is increased, and the cells switch to a nonproliferative state. **B** WTAP in the antiviral immune response. WTAP is degraded in virus-infected cells. After viral infection, degradation of WTAP leads to a decrease in the m^6^A level of IRF3 mRNA and IFNAR1 mRNA, which leads to IRF3 mRNA translation blockade and accelerated IFNAR1 mRNA degradation. This biological process restricts the antiviral immune response and maintains homeostasis.

WTAP regulates CDK2 mRNA stability, which is related to the G1/S transition [88], in renal cell carcinoma (RCC) and keratinocytes [89]. During RCC cell proliferation, WTAP enhances the stability of the CDK2 mRNA by directly binding to its 3ʹ-UTR (Fig. 2) [89]. In psoriasis, WTAP not only stabilizes the CDK2 mRNA but also stabilizes the cyclin-A2 mRNA, which promotes the G2/M transition [90]. The binding motif of WTAP in the cyclin-A2 mRNA is ACAAAAUUAU (1526–1534) [82]. Smooth muscle cells (SMCs) proliferate during vascular restructuring and switch to a nonproliferative state when remodeling is complete [91]. The efficiency of WT1 binding to its target promoter is affected by WTAP in the nucleus. Amphiregulin belongs to the epidermal growth factor gene family, which serves as a strong mitogen in SMCs and is regulated by WT1 [92]. When WTAP levels decrease in SMCs, more WT1 bound to the promoter of amphiregulin, switching the cell to a proliferative state. Bcl-2, a protooncogenic apoptosis suppressor, is also activated by WT1 [93]. WTAP was upregulated when SMCs were in a nonproliferative state or the late stage of repair in the intima of injured arteries. Overexpression of WTAP prevents WT1 from binding to the Bcl-2 promoter, thereby downregulating Bcl-2 and activating apoptosis (Fig. 3A) [94].

#### pre-mRNA splicing

Alternative splicing of pre-mRNAs plays important roles in cell differentiation and development, and recent studies indicated that most human multiexon genes exhibit alternative splicing [8]. If this process is not highly regulated and accurate, it will lead to mis-splicing events, which may result in proteins with altered function [95].

WTAP interacts with the nuclear splicing factor WT1, forming a splicing complex [96]. Female-specific regulatory protein sex-lethal (SXL) affects sex-specific splicing by regulating the female-specific splicing of *transformer (tra)* pre-mRNA. Moreover, FL (2)D, the Drosophila homolog of WTAP, forms an RNA-independent complex with SXL [97]. When Fl(2)D was immunodepleted, alternative splicing of *transformer* pre-mRNA, the target of SXL regulation, was affected [98].

In Drosophila, FL(2)d is distributed throughout the entire eye-antennal imaginal disc and affects retinal development [96] by regulating the alternative splicing of the eye developmental gene Ultrabithorax (Ubx) [99]. In mammalian cells, WTAP and its complex (VIRMA, CBLL1, and ZC3H13) regulate alternative splicing and alternative polyadenylation via inhibitory mechanisms in GC-rich sequences [100].

Furthermore, WTAP was found in complexes related to splicing factors, including Snf, U170k, and the two U2AF subunits U2AF38 and U2AF50 [97]. In conclusion, WTAP is closely related to pre-mRNA splicing, but its specific role in this process remains unclear.

#### Antiviral responses

WTAP is degraded in virus-infected cells through the K48-linked ubiquitination-proteasome pathway upon activation of type I interferon (IFN-I) signaling. IFN-regulatory factor 3 (IRF3) and interferon-alpha/beta receptor subunit 1 (IFNAR1) are two key components involved in IFN-I signaling that are regulated by WTAP in an m^6^A-dependent manner. WTAP maintains the expression of IRF3 and IFNAR1 by enhancing IRF3 translation efficiency via m^6^A modification at its 5’UTR and improving IFNAR1 mRNA stability via m^6^A modification at its 3’UTR at the same time. Following viral infection, degradation of WTAP blocks IRF3 mRNA translation and accelerates IFNAR1 mRNA degradation, which restricts the antiviral immune response and maintains homeostasis (Fig. 3B) [101].

## Expression of WTAP in cancers

**Fig. 4:**
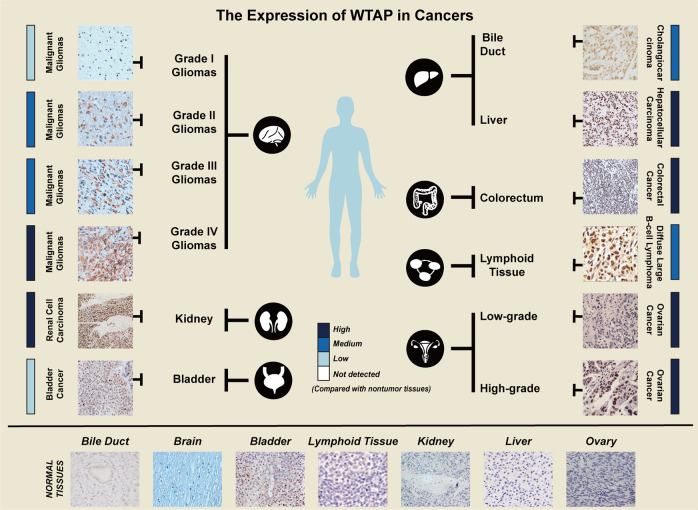
The function of WTAP in biological process. Immunohistochemistry has been performed in many studies. Strong staining for WTAP was observed in grade IV gliomas, renal cell carcinoma, hepatocellular carcinoma, colorectal cancer, and high-grade ovarian carcinoma, with low staining in adjacent normal tissues.

In patient tissue samples, immunohistochemistry results and western blot results have shown that WTAP is highly expressed in dozens of cancers (Fig. 4 Table 2).

**Table 2:** WTAP expression in different cancers.

Cancer	Expression	Role	References
Hepatocellular carcinoma	Upregulated	Oncogene	[105]
Osteosarcoma tumorigenesis	Upregulated	Oncogene	[106]
Gastric cancer	Upregulated	Oncogene	[107]
Acute myeloid leukemia	Upregulated	Oncogene	[114, 132]
Natural killer/T-cell lymphoma	Upregulated	Oncogene	[123]
Cholangiocarcinoma	Upregulated	Oncogene	[126]
Diffuse large B-cell lymphoma	Upregulated	Oncogene	[134]
Malignant glioma	Upregulated	Oncogene	[135]
Colorectal cancer	?	Tumor Suppressor	[137]
Pancreatic ductal adenocarcinoma	Upregulated	Oncogene	[142]
Bladder cancer	Upregulated	Oncogene	[143]
Renal cell carcinoma	Upregulated	Oncogene	[89]
High-grade serous ovarian cancer	Upregulated	Oncogene	[145]
Non-small cell lung cancer	?	Oncogene	[147]

## WTAP as an m^6^A methyltransferase in cancer

### WTAP in hepatocellular carcinoma (HCC)

**Fig. 5:**
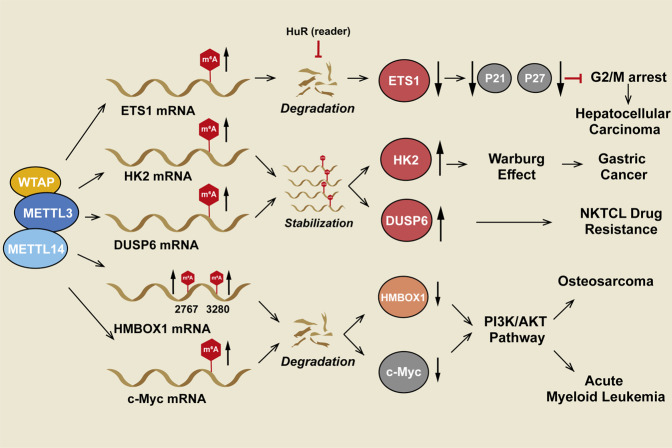
WTAP serves as a methyltransferase in cancers. WTAP plays a significant role in RNA methylation by recruiting METTL3/METTL14 to form a complex that binds to target RNAs. In this process, WTAP regulates the differential expression of oncogenes and tumor suppressor genes in an m^6^A-dependent manner. It enhances the stability of the HK2 and DUSP6 mRNAs, inducing drug resistance in hepatocellular carcinoma, gastric cancer, and NKTCL. Additionally, WTAP induces the degradation of the ETS1, HMBOX1, and c-Myc mRNAs in an m^6^A-dependent manner, enhancing HCC proliferation and suppressing the invasion and metastasis of osteosarcoma and acute myeloid leukemia.

The overexpression of WTAP was found to be correlated with a poor prognosis in HCC, and WTAP expression promoted proliferation and metastasis in vitro and vivo [102]. ETS1 is a transcriptional activator that is typically regulated by the Ras/Raf/MEK/ERK pathway [103], and it serves as a tumor suppressor in HCC by downregulating the transcription of p21 and p27 [102]. The expression of ETS1 is regulated by HuR, an RNA-binding protein that binds to and stabilizes m^6^A-modified RNA [104], and WTAP. WTAP was confirmed to increase the m^6^A modification of ETS1 mRNA and interfere with the interaction between ETS1 mRNA and HuR. Thus, WTAP downregulates p21 and p27 expression to promote HCC proliferation (Fig. 5, Table 3) [102, 105].

**Table 3:** WTAP as an m6A methyltransferase in cancer.

Cancer	Biological function	Mechanism	Target	Regulator	References
Hepatocellular carcinoma	Enhance proliferation, migration	Downregulated the ETS1/p21, p27 axis in an m6A-mediated manner	ETS1/p21, p27	/	[105]
Osteosarcoma tumorigenesis	Enhance proliferation, migration	Downregulated the HMBOX1/PI3K/AKT axis in an m6A-mediated manner	HMBOX1/PI3K/AKT	/	[106]
Gastric cancer	Enhance proliferation, migration	WTAP enhanced the stability of HK2 mRNA to regulate the gastric cancer Warburg effect	HK2	/	[107]
Acute myeloid leukemia	Enhance proliferation	Performed m6A on c-Myc mRNA and enhanced its degradation	c-Myc	Cyclins and Hsp90	[114, 132]
Natural killer/T-cell lymphoma	Promote resistance to cisplatin	Enhanced m6A on DUSP6 and stabilized its mRNA	DUSP6	/	[123]

### WTAP in osteosarcoma

WTAP was found to be highly expressed in osteosarcoma, and it was a significant independent prognostic factor for overall survival [106]. Chen et al. found that upregulation of WTAP reduces the expression of HMBOX1, an oncogene that inhibits osteosarcoma proliferation and metastasis by downregulating the PI3K/AKT pathway. Specifically, WTAP regulated HMBOX1 in an m^6^A-dependent manner. The m^6^A modification sites in HMBOX1 are in the 3ʹUTR at 2767 and 3080 nucleotides. However, the reader of HMBOX1 m^6^A remains unclear (Fig. 5, Table 3) [106].

### WTAP in gastric cancer

WTAP was found to be highly expressed in gastric cancer tissues, and its overexpression was correlated with poor prognosis [107]. HK2 plays significant roles in both the Warburg effect, a significant cause of relapse and pathogenesis in gastric cancer [108], and cancer cell immortalization [109]. WTAP promoted the proliferative ability of gastric cancer cells and increased their glycolytic capacity (glucose uptake, lactate production, and extracellular acidification rate) by stabilizing the hexokinase-2 (HK2) mRNA by binding to its 3ʹ-UTR m^6^A site (Fig. 5, Table 3) [107].

### WTAP in hematological malignancies

WTAP was overexpressed in acute myeloid leukemia (AML) patients, and its expression was related to a poor survival rate. MYC is known as a master transcription factor that regulates genes essential for survival, cell proliferation, and metastasis [110, 111] and may act as a downstream regulator of the PI3K/AKT pathway [112, 113]. WTAP downregulates c-Myc expression by increasing the m^6^A modification of its mRNA [114]. Thus, high WTAP expression predicts poor prognosis in AML, and WTAP plays an epigenetic role in AML (Fig. 4, Table 2) [114].

It was also reported that PIWI-interacting RNAs (piRNAs) are related to diffuse large B-cell lymphoma (DLBCL) [115]. piRNA 30473 was highly expressed in DLBCLs, where it promoted proliferation and induced cell cycle arrest. Mechanistically, piRNA-30473 increased WTAP levels to upregulate the global m^6^A level. WTAP increased HK2 expression by enhancing its m^6^A level. The m^6^A reader IGF2BP2 was found to bind to the 5ʹUTR of HK2 mRNA, leading to its stabilization. HK2 is an essential kinase in glucose metabolism that is associated with tumor cell proliferation by enhancing aerobic glycolysis [116–119]. Overall, the piRNA-30473/WTAP/HK2 axis contributes to tumorigenesis by regulating m^6^A RNA methylation in DLBCL [115] (Fig. 5, Table 3).

Natural killer/T-cell lymphoma (NKTCL) exhibits high resistance to chemotherapy, which is related to the high expression of ATP binding cassette (ABC) transporter proteins as drug efflux pumps [120, 121]. Multidrug resistance-associated protein 1 (MRP1) and P-glycoprotein (P-gp) are two major proteins in the ABC transporter family that prevent the cellular accumulation of chemotherapy drugs [122]. WTAP was upregulated in NKTCL cell lines. Depletion of WTAP downregulated the expression of MRP1 and P-gp and blocked resistance to cisplatin [122, 123]. WTAP also upregulated the expression of dual-specificity phosphatase 6 (DUSP6) by stabilizing its mRNA by increasing the m^6^A modification of its transcript, which induced tumor progression and contributed to WTAP-induced drug resistance via the WTAP/m^6^A/DUSP6 axis (Fig. 5, Table 3) [123].

### WTAP in endometrial carcinoma (EC)

WTAP was observed to be upregulated in endometrial cancer cell lines [124, 125]. WTAP activated the nuclear factor‐κB (NF‐κB) pathway by regulating the m^6^A modification of caveolin‐1 (CAV‐1) mRNA. Reduction of CAV-1 levels by WTAP could enhance the activity of the NF‐κB pathway, contributing to the pathogenesis of EC [124, 125].

## Other functions of WTAP in cancer

### WTAP in cholangiocarcinoma

**Fig. 6:**
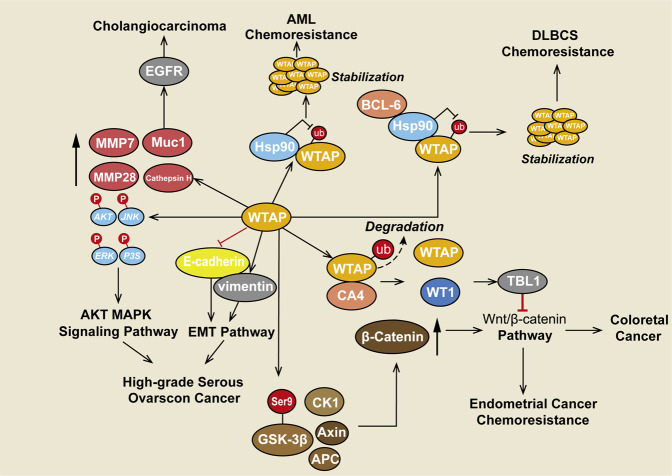
Other functions of WTAP in cancers. WTAP regulates the differential expression of oncogenes and tumor suppressor genes at the non-posttranscriptional level. WTAP induces the expression of Muc1, which regulates EGFR activity in cholangiocarcinoma. Hsp90 forms a complex with WTAP and stabilizes its protein level to promote chemoresistance in AML. In DLBCL, Hsp90 also stabilizes the WTAP protein, which forms a complex with BCL6. In colorectal cancer, CA4 interacts with WTAP and promotes its degradation in a polyubiquitination-dependent manner so that WT1 is released from the WT1-WTAP complex, resulting in the induction of transducin β-like protein 1 (TBL1) and the degradation of β-catenin, which blocks the Wnt pathway. WTAP was found to facilitate the nuclear translocation of β-catenin and enhance the phosphorylation of GSK3b at Ser9, which induced chemoresistance to cisplatin in endometrial carcinoma by activating the Wnt/β-catenin pathway. Additionally, WTAP was found to regulate the expression of the EMT-related proteins E-cadherin and vimentin. Furthermore, WTAP is involved in the activation of the AKT and MAPK pathways. Overall, WTAP contributes to cell proliferation, apoptosis, invasion, metastasis, and chemo- or radioresistance in different cancers.

WTAP shows a tendency toward overexpression in cholangiocarcinoma tissues. In addition, overexpression of WTAP induces the expression of MMP7, MMP28, cathepsin H, and Muc1 [126]. Notably, these enzymes are all involved in the degradation of the extracellular matrix, which can explain the increased invasion of cholangiocarcinoma cells and WTAP overexpression inside lymph nodes or vessels [127–130]. In addition, Muc1 was shown to regulate EGFR activity [131] to regulate the motility of cancer cells [126]. Therefore, the function of WTAP is an important in cholangiocarcinoma (Fig. 6, Table 4).

**Table 4:** Other functions of WTAP in cancer.

Cancer	Biological function	Mechanism	Target	Regulator	References
Cholangiocarcinoma	Promote invasion, migration	/	MMP7, MMP28, Cathepsin H, Muc1	/	[126]
Diffuse large B-cell lymphoma	Promote proliferation, counteract etopside-mediated apoptosis	/	/	Cyclins and Hsp90	[134]
Colorectal cancer	/	WTAP supports CA4 in performing its tumor-suppressive function and releasing WT1 from the WTAP-WT1 complex	Carbonic anhydrase IV (CA4)	/	[137]
Renal cell carcinoma	Promote invasion proliferation and migration, accelerate cell cycle progression	Binds to the CKD2 transcript to enhance the function of its mRNA	/	/	[89]
High-grade serous ovarian cancer	Proliferation, migration and inhibition of apoptosis abilities	Regulates the epithelial-mesenchymal transition (EMT) pathway and AKT and MAPK signaling pathways	E-cadherin, Vimentin, AKT, JNK, ERK and p38	/	[145]
Non-small cell lung cancer	Proliferation, migration and inhibition of apoptosis abilities	/	/	PCGEM1/miR-433–3p axis	[147]

### WTAP in hematological malignancies

In AML, the molecular chaperone Hsp90 interacted with and stabilized WTAP by decreasing its polyubiquitination, which promoted chemoresistance (Fig. 5, Table 3) [132]. This phenomenon was also observed in diffuse large B-cell lymphoma (DLBCL), a common type of non-Hodgkin lymphoma [133, 134] (Fig. 6, Table 4).

### WTAP in malignant glioma

WTAP is overexpressed in glioma tissues compared to normal brain tissues. Furthermore, WTAP expression is associated with glioma grade and is an independent prognostic factor for shorter survival in patients with glioma. High expression of WTAP leads to a much lower overall survival rate than low WTAP expression in patients suffering from glioma. Therefore, WTAP may be a novel prognostic marker for glioma (Table 4) [135].

### WTAP in endometrial carcinoma (EC)

WTAP also promoted chemoresistance of endometrial carcinoma (EC) cells to cisplatin by facilitating proliferation and repressing apoptosis. Mechanistically, WTAP enhanced the phosphorylation of GSK3β at Ser9, which facilitated the nuclear translocation of β-catenin [136]. Consequently, β-catenin activated the transcription of c-Myc, Survivin, and Bcl-xl to promote chemoresistance to cisplatin [136]. Overall, these results shed light on the strategies to modify the treatment response by altering chemoresistance to cisplatin (Fig. 6 Table 4) [124].

### WTAP in colorectal cancer (CRC)

Carbonic anhydrase IV (CA4) is silenced in colorectal cancer (CRC) [137]. It was recently identified as a preferentially methylated gene that is expressed in normal colon tissues [138] and plays a tumor-suppressive function by inhibiting the Wnt/β-catenin signaling pathway [139, 140]. CA4 interacts with WTAP and promotes its polyubiquitination-dependent degradation [137]. WT1 is a negative regulator of the Wnt signaling pathway [141]. WT1 is released from the WT1-WTAP complex by CA4, resulting in the induction of transducing β-like protein 1 (TBL1) and the degradation of β-catenin. A lack of CA4 results in the activation of WNT/β-catenin signaling, which promotes CRC progression [137] (Fig. 6, Table 4).

### WTAP in pancreatic ductal adenocarcinoma (PDAC)

The nuclear and cytoplasmic levels of WTAP were much higher in PDAC than in adjacent nontumor tissues [142]. High nuclear levels of WTAP were correlated with a more advanced tumor stage, while cytoplasmic WTAP levels were associated with histological trade and perineural invasion. In addition, high expression of WTAP in the nucleus and cytoplasm differed significantly by sex. Nuclear WTAP levels were identified as an independent prognostic indicator for PDAC and were associated with poor overall survival. Overall, WTAP may be a molecular biomarker in PDAC [142] (Table 4).

### WTAP in bladder cancer

Immunohistochemical staining showed that WTAP expression in bladder cancer was significantly higher than that in normal tissues, and high expression of WTAP indicated a poor prognosis [143]. Moreover, both the mRNA and protein levels of WTAP were upregulated in bladder cancer, offering a potential novel approach for the diagnosis and treatment of bladder cancer (Table 4) [143].

### WTAP in renal cell carcinoma (RCC)

In RCC, WTAP binds to the transcript of CDK2, a cell cycle-related protein [144], to enhance the stability of its mRNA, thus decreasing the percentage of cells in the G1 phase (Table 4) [89].

### WTAP in high-grade serous ovarian cancer (HGSOC)

WTAP expression was correlated with a poor prognosis in high-grade serous ovarian cancer (HGSOC) [145]. Mechanistically, WTAP affected migration by regulating proteins related to the epithelial-mesenchymal transition (EMT) by decreasing E-cadherin expression and increasing vimentin expression. In addition, WTAP promoted the phosphorylation of AKT, JNK, ERK, and p38, indicating that WTAP might be involved in activation of the AKT and MAPK signaling pathways (Fig. 6, Table 4) [145].

It was also reported that family with sequence similarity 76-member A (FAM76A) and HBS1-like translational GTPase (HBS1L) are positively correlated with WTAP according to weighted gene coexpression network analysis (WGCNA), and both were correlated with a poor prognosis [146].

### WTAP in non-small cell lung cancer (NSCLC)

High levels of the lncRNA PCGEM1, which is considered to promote cell growth, were detected in NSCLC. PCGEM1 was mostly distributed in the cytoplasm, indicating that it mostly performs its function at the posttranscriptional level. Furthermore, PCGEM1 was found to act as a sponge for miR-433–3p in NSCLC. WTAP is a downstream target of the PCGEM1/miR-433-3p axis. Overall, PCGEM1 plays an important role in NSCLC and can accelerate cancer progression via the miR-433-3p/WTAP axis (Table 4) [147].

### WTAP in hepatoblastoma

Hepatoblastoma is a common primary malignant hepatic tumor of infancy and childhood that usually occurs in the first two years of life [148]. Hepatoblastoma susceptibility was correlated with WTAP gene variants. The genotype frequencies of three WTAP single nucleotide polymorphisms (SNPs: rs7766006 G > T, rs9457712 G > A, and rs1853259 A > G) were evaluated in Chinese children, including 313 hepatoblastoma patients and 1446 controls. However, only the rs7766006 GT/TT genotype exhibited a significant association with hepatoblastoma risk. Rs7766006 T was associated with a decrease in WTAP mRNA levels. Thus, WTAP SNPs potentially play a role in hepatoblastoma via genetic modification [149].

## Future prospects

WTAP was first reported to be a splicing factor. In the following years, its biological functions have gradually been uncovered, including functions in m^6^A modification, embryo development, cell cycle progression and differentiation, pre-mRNA splicing, and antiviral responses. With the development of techniques for detecting m^6^A modification, WTAP was revealed to be a part of the MTC and to participate in m^6^A modification with both METTL3 and METTL14 and other methyltransferases. In human umbilical vein endothelial cells, WTAP promotes G2/M transition, while in smooth muscle cells, overexpression of WTAP prevents WT1 from binding to the Bcl-2 promoter, thereby downregulating Bcl-2 and activating apoptosis. In renal cell carcinoma, keratinocytes, and psoriasis, WTAP regulates the G1/S transition and G2/M transition by stabilizing specific mRNAs. Thus, WTAP may be a potential biomarker for changes in cell proliferation and differentiation. WTAP is also associated with chemoresistance in hematological malignancies and endometrial carcinoma by upregulating the expression of MRP1 and P-gp and enhancing the phosphorylation of GSK3β at Ser9. These results shed light on the potential of targeting WTAP for the prevention of chemoresistance to cisplatin. During metabolism, WTAP can stabilize the HK2 mRNA, which is associated with aerobic glycolysis and the Warburg effect in diffuse large B-cell lymphoma. The therapeutic schedule can be developed according to this metabolic phenomenon. High expression of WTAP was confirmed in malignant gliomas, renal cell carcinoma, hepatocellular carcinoma, colorectal cancer, and ovarian cancer, which is related to progression and poor prognosis (Fig. 6, Table 4), suggesting that WTAP might be a biomarker for the above cancers. In liver cancer, WTAP was observed to increase the m^6^A level of the ETS1 mRNA, thereby facilitating cancer progression. Similarly, WTAP was found to induce the proliferation and metastasis of osteosarcoma by regulating HMBOX1 m^6^A modification. In gastric cancer, WTAP enhanced HK2 mRNA stability through m^6^A modification. In natural killer/T-cell lymphoma, WTAP upregulated DUSP6 expression through m^6^A modification, inducing drug resistance. In acute myeloid leukemia, WTAP downregulated c-Myc expression by increasing the m^6^A modification of its mRNA, making cells resistant to chemotherapy drugs. These cases indicated that the role of WTAP as a methyltransferase is vital in cancer progression. Although no small-molecule inhibitors of RNA methyltransferases and WTAP have been discovered, FTO demethylation inhibitors have been identified. Rhein can bind the FTO catalytic domain to suppress m^6^A demethylation [150]. CHTB, N-CDPCB and meclofenamic acid 2 (MA2) have been revealed to be FTO inhibitors through structure-based virtual screening and biochemical analyses [151, 152]. R-2-hydroxyglutarate (R-2HG) inhibits FTO activity and increases global m^6^A modification, which has been tested in vitro and in mice [153]. These effects suggest that WTAP-targeted inhibitors may be developed in the future and that a deeper understanding of m^6^A modification is warranted.

## Conclusion

At present, our understanding of WTAP is insufficient due to a lack of further experiments and additional samples. m^6^A has gradually become a significant focus of cancer research, but the role of WTAP in this process is still at an early stage. Furthermore, the localization of WTAP in nuclear speckles and the formation of a complex with METTL3 and METTL14 need to be further investigated, since this knowledge may be useful for understanding the role of m^6^A modification in cancer biology. In conclusion, many studies have revealed WTAP as a potential biomarker for predicting cancer progression, since it participates in alternative splicing, cell cycle regulation and methylation. Thus, efforts should be made to develop the potential of WTAP for therapies targeting tumorigenesis and tumor development.

## Data Availability

The materials that support the conclusion of this review have been included within the article.
